# Use of the senolytics dasatinib and quercetin for prevention of pelvic organ prolapse in a mouse animal model

**DOI:** 10.18632/aging.206120

**Published:** 2024-09-26

**Authors:** Erryn Tappy, Haolin Shi, Jessica Pruszynski, Maria Florian-Rodriguez

**Affiliations:** 1 University of Texas Southwestern Medical Center, Department of Obstetrics and Gynecology, Dallas, TX 75390, USA

**Keywords:** pelvic organ prolapse, cellular senescence, senolytic agents, animal model

## Abstract

Objective: Senolytic agents have the potential to target age-related pathology associated with cellular senescence and reduce senescent cell activity in several disease processes. We utilized a mouse model of pelvic organ prolapse, Fibulin-5 knockout (*Fbln-5^-/-^)* mice, to assess the ability of dasatinib and quercetin (D+Q) to prevent development of prolapse.

Methods: Four-week-old female *Fbln-5^-/-^* (n=63) and wild-type (WT) mice (n=54) were assigned to control (vehicle injection) or treatment (D = 5 mg/kg, Q = 50 mg/kg) groups. Weekly oral gavage injections were administered from weeks 4-8 of life. Pelvic organ prolapse quantification system measurements were obtained weekly. Vaginal tissue was harvested at 10, 12 and 20 weeks. Tissue analysis included immunostaining for cell cycle inhibitors, multiplex cytokine analysis, senescence-associated-β-galactosidase (SA-β-Gal) and histologic analysis of extracellular matrix proteins.

Results: Perineal body length was significantly longer in *Fbln-5^-/-^* treatment mice at 20 weeks. Expression of p16 and p53 was decreased in *Fbln-5^-/-^* treatment mice compared to controls (4.0% vs. 26.7%, p=0.0124 and 2.9% vs. 16.8%, p=0.272) at 20 weeks. Expression of SA-β-Gal and senescence-associated cytokines did not vary significantly between groups. At 20 weeks, vaginal tissue elastin content in *Fbln-5^-/-^* treatment mice increased compared to controls (1.04% vs. 0.84%, p=0.999).

Conclusions: D+Q injections did not result in clinically significant differences in prolapse development but did demonstrate decreased expression of cellular senescence markers in *Fbln-5^-/-^* mice. This suggests senolytic agents may mitigate contributions of cellular senescence to tissue dysfunction associated with prolapse. Further studies are needed to confirm ideal timing, dosage, and route of senolytics in prevention of prolapse.

## INTRODUCTION

Pelvic organ prolapse is a highly prevalent condition among women in the United States. The current estimated risk of undergoing surgery for pelvic organ prolapse is 13% by the age of 80 [1–3]. Two well known risk factors for developing prolapse are age and vaginal delivery, but the underlying cellular mechanisms that ultimately cause prolapse in response to these factors are not yet well elucidated [4, 5]. Recent studies have demonstrated a process known as cellular senescence may be a potential contributing factor [6–9]. Senescence is a state of irreversible growth arrest that occurs in response to a variety of cellular stressors such as DNA damage, oxidative stress, telomere erosion, and tumorigenesis [10]. Senescence renders cells resistant to apoptosis and stimulates secretion of pro-inflammatory cytokines, chemokines, pro-thrombotic factors, and extracellular matrix proteases that cause tissue damage and dysfunctional tissue architecture or fibrosis [11]. This secretory state is called the senescence-associated secretory phenotype (SASP). Senescent cells accumulate during aging and have been linked to a variety of age-related diseases such as atherosclerosis, heart failure, pulmonary hypertension, diabetes mellitus and osteoarthritis [12–15].

Studies in both aging and pregnant mice have demonstrated cellular changes consistent with those induced by cellular senescence. Fibulin-5 knockout mice (*Fbln-5^-/-^*) are genetically deficient in elastic fiber assembly and develop pelvic organ prolapse as a function of age. Increased secretion of SASP proteases (MMP2 and MMP9), which are capable of degrading connective tissue elastic fibers, occurs just prior to the onset of prolapse in the vaginal tissue of *Fbln-5^-/-^* mice, around 4 weeks of age [6]. Subsequent to vaginal distention simulating vaginal birth, secretion of senescent markers p53 and γ-H2Ax increases in the vaginal tissue of *Fbln-5^-/-^* mice compared to wild type controls [8]. Prolapse also occurs earlier in *Fbln-5^-/-^* mice undergoing distention compared to non-distended mice [7, 8]. Collectively these findings suggest senescence is correlated with the underlying pathogenesis of prolapse development associated with age and injury related to vaginal birth.

Attempts to ameliorate the effects of cellular senescence on age-related disease have been made via development of senolytic agents, which target and eliminate senescent cells. The combination of senolytic agents dasatinib and quercetin has been shown to decrease the senescent cell burden in diabetic kidney disease and pulmonary fibrosis [16, 17]. An *in vitro* study of rat vaginal fibroblast cells demonstrated that fibroblasts exposed to etoposide undergo cellular senescence, and treatment with dasatinib and quercetin (D+Q) results in clearance of these senescent cells [9]. This led us to consider the ability of D+Q to clear senescent cells in the vaginal tissue of *Fbln-5^-/-^* mice. We hypothesize that *Fbln-5^-/-^* vaginal tissue undergoes cellular senescence, and senolytics can reduce senescent cell burden, potentially delaying the onset of prolapse or reducing prolapse severity.

## RESULTS

### Evaluation of clinical development of pelvic organ prolapse

**Figure 1:**
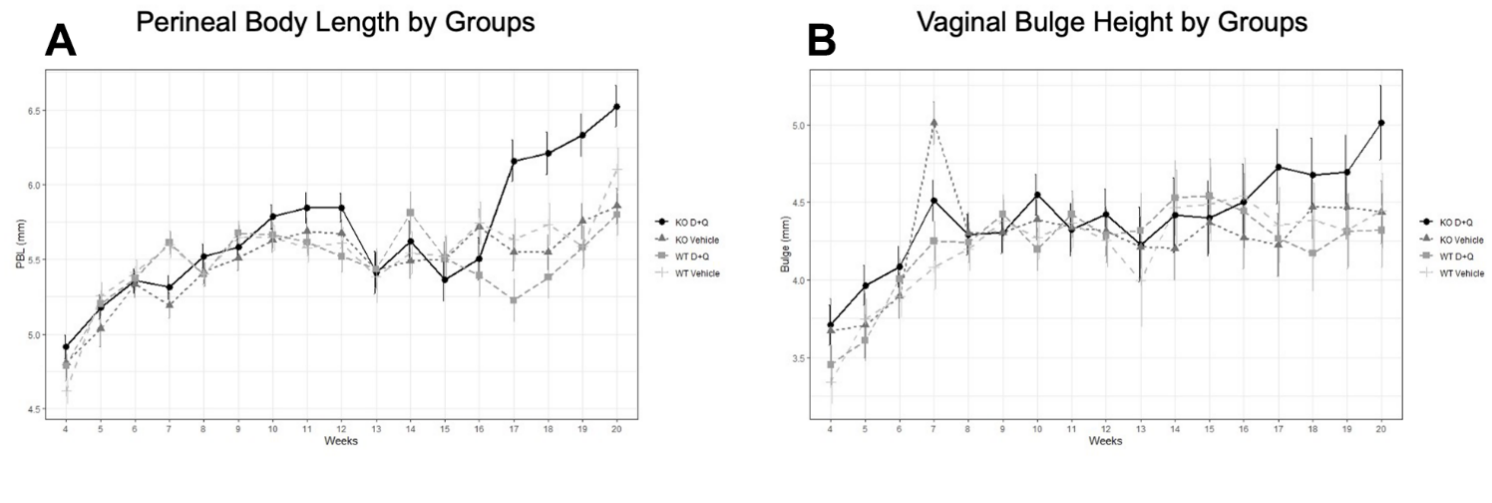
**Impact of serial dasatinib and quercetin (D+Q) injections on development of pelvic organ prolapse in wild type (WT) and Fibulin-5 knockout (*Fbln-5^-/-^*) mice.** D+Q or placebo injections were administered at weeks 4, 5, 6, 7 and 8 of life to treatment and control groups, respectively. Mouse pelvic organ prolapse quantification measurements were used to obtain weekly perineal body length (**A**) and vaginal bulge height (**B**) values. WT control (n=27), WT treatment (n=27), *Fbln-5^-/-^* control (n=33), *Fbln-5^-/-^* treatment (n=30). p <0.05 between *Fbln-5^-/-^* treatment mice and *Fbln-5^-/-^* control mice, and between *Fbln-5^-/-^* treatment mice and WT treatment mice (**A**).

MOP-Q assessments were used to determine the impact of serial injections of D+Q on the development of pelvic organ prolapse in *Fbln-5^-/-^* and WT mice beginning at 4 weeks of age. Perineal body length (PBL) and vaginal bulge height gradually increased with increasing age and weight of *Fbln-5^-/-^* and WT mice (Figure 1). No significant differences between WT mice in the treatment and control groups were seen. Beginning at 18 weeks, *Fbln-5^-/-^* mice in the treatment group had significantly larger PBL measurements compared to *Fbln-5^-/-^* mice in the control group. At 18 weeks of age, the mean PBL in *Fbln-5^-/-^* control mice compared to mice in the *Fbln-5^-/-^* treatment group was 5.55mm vs. 6.21mm (p=0.0019 95% CI[0.19, 1.14]), at 19 weeks was 5.75mm vs. 6.21mm (p=0.0094, 95% CI[0.10,1.05]) and at 20 weeks was 5.86mm vs. 6.53mm (p=0.0017, 95% CI[0.19, 1.14]), respectively. Only one mouse in the *Fbln-5^-/-^* treatment arm developed Stage 2 prolapse, with the remaining having either Stage 0 or Stage 1, which was seen in two animals. Significant differences were also seen in PBL between *Fbln-5^-/-^* mice in the treatment arm compared to WT mice in the treatment arm beginning at 18 weeks of age. Vaginal bulge heights were not significantly different between *Fbln-5^-/-^* treatment or control mice.

### Evaluation of senescence markers

To determine if administration of the senolytic agents D+Q modified the expression of markers of cellular senescence, vaginal tissue was first immunostained for the cell cycle inhibitors, p16, p53 and p21. The mean percent vaginal tissue area immunostained for p16, p53 and p21 at 10, 12 and 20 weeks of age is shown in Table 1 for WT and *Fbln-5^-/-^* treatment and control groups.

**Table 1:** Percentage of mouse vaginal tissue stained for p16, p53 and p21 immunofluorescence.

**Week**	**WT control**	**WT treatment**	***Fbln5^-/-^* control**	***Fbln5^-/-^* treatment**	**p-value within WT**	**p-value within *Fbln5^-/-^***	**p-value within WT and *Fbln5^-/-^* D+Q groups**
p16							
10	9.14	10.33	3.67	14.96	>0.99 [-19.48, 21.84]	0.77 [-9.37, 31.96]	0.99 [-16.03, 25.30]
12	5.18	15.55	12.26	2.34	0.85 [-10.30, 31.03]	0.88 [-30.59, 10.74]	0.56 [-33.87, 7.46]
20	4.16	6.79	26.65	3.94	>0.99 [-18.03, 23.30]	**0.012** [-42.49, -2.92]	>0.99 [-22.63, 16.93]
p53							
10	2.95	2.12	2.72	4.01	>0.99 [-19.42, 17.76]	>0.99 [-15.68, 21.50]	>0.99 [-16.71, 20.47]
12	9.54	3.02	9.54	6.70	0.99 [-25.10, 12.08]	>0.99 [-15.68, 21.50]	0.99 [-14.91, 22.27]
20	4.92	3.07	16.79	2.94	>0.99 [-20.44, 16.74]	0.27 [-31.64, 3.95]	>0.99 [-17.93, 17.67]
p21							
10	0.26	0.67	0.75	2.14	>0.99 [-4.48, 5.30]	0.99 [-3.49, 6.28]	0.17 [-0.70, 8.65]
12	0.50	0.69	0.85	0.59	>0.99 [-4.70, 5.07]	>0.99 [-5.15, 4.62]	>0.99 [-4.99, 4.78]
20	1.10	0.75	0.50	4.73	>0.99 [-5.24, 4.53]	0.11 [-0.45, 8.91]	0.99 [-3.42, 6.35]

Data are marginal means and p-values with 95% CI. Bolded values indicate statistical significance. WT control (n=27), WT treatment (n=27), *Fbln-5^-/-^* control (n=33), *Fbln-5^-/-^* treatment (n=30).

**Figure 2:**
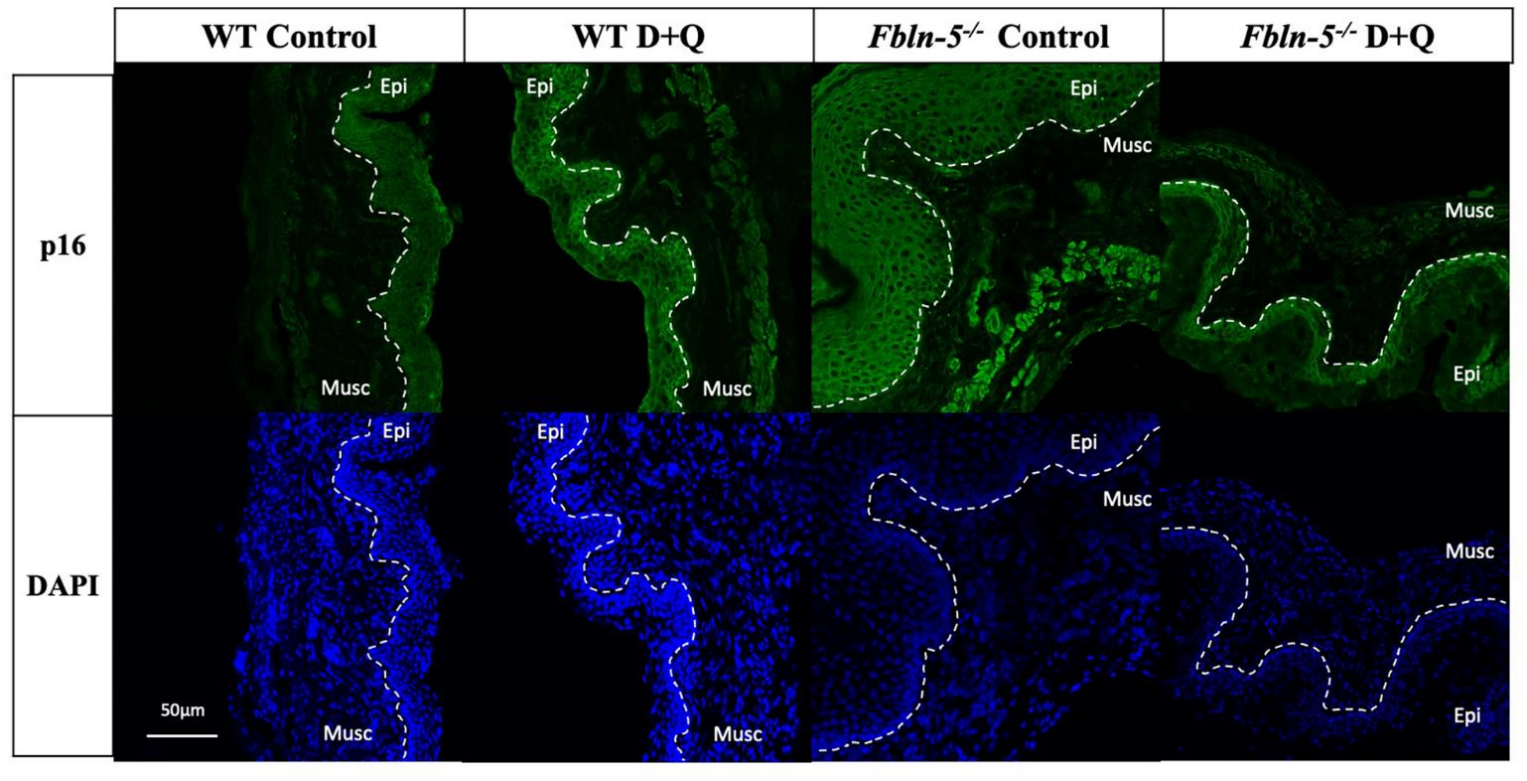
**Impact of serial dasatinib and quercetin (D+Q) injections on p16 expression in the vaginal tissue of wild type (WT) and Fibulin-5 knockout (*Fbln-5^-/-^*) mice at 20 weeks of life.** Representative tissue sections from WT control, WT treatment, *Fbln-5^-/-^* control and *Fbln-5^-/-^* treatment mice. Top panels demonstrate differences in p16 expression between groups. Bottom panels display corresponding DAPI staining to label cellular nuclei for anatomical reference. Epi=epithelium, Musc=muscularis. 20x magnification.

p16 immunoreactivity was seen in cells both within the vaginal epithelium and vaginal muscularis layer in *Fbln-5^-/-^* and WT mice vaginas (Figure 2). In *Fbln-5^-/-^* control mice, p16 immunostaining increased over time and comprised 3.7% of vaginal tissue at 10 weeks of age, 12.3% at 12 weeks and 26.7% at 20 weeks. At 20 weeks of age, p16 immunostaining was significantly higher in the *Fbln-5^-/-^* mice control group as compared to the *Fbln-5^-/-^* treatment group (4.0% of the vaginal tissue). Significant differences were seen in p16 expression at 20 weeks between the *Fbln-5^-/-^* control group as compared to the WT control group (26.7 vs. 4.6, p=0.022, 95% CI [1.82, 43.2]). No significant differences were seen in p16 expression between the WT control and treatment groups at all time points analyzed.

**Figure 3:**
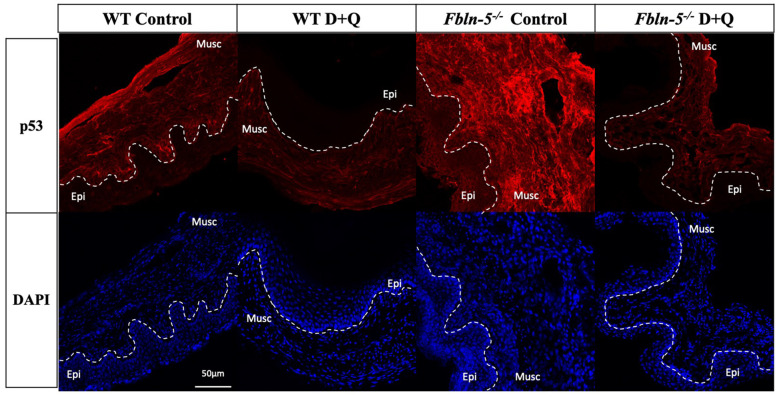
**Impact of serial dasatinib and quercetin (D+Q) injections on p53 expression in the vaginal tissue of wild type (WT) and Fibulin-5 knockout (*Fbln-5^-/-^*) mice at 20 weeks of life.** Representative tissue sections from WT control, WT treatment, *Fbln-5^-/-^* control and *Fbln-5^-/-^* treatment mice. Top panels demonstrate differences in p53 expression between groups. Bottom panels display corresponding DAPI staining to label cellular nuclei for anatomical reference. Epi=epithelium, Musc=muscularis. 20x magnification.

Immunostaining for cell cycle inhibitor p53 was seen in both the vaginal epithelium and vaginal muscularis layer of *Fbln-5^-/-^* and WT mice vaginas, though with much greater intensity in the muscularis layer (Figure 3). p53 immunostaining displayed a similar trend to that of p16 in which staining in the *Fbln-5^-/-^* control group increased at each time point. At 20 weeks p53 staining was present in 16.8% of vaginal tissue in *Fbln-5^-/-^* control mice as compared to 2.94% of *Fbln-5^-/-^* mice in the D+Q group, though this difference did not reach statistical significance. No significant differences were seen between the *Fbln-5^-/-^* and WT treatment and control groups, nor within WT control and treatment groups at all time points analyzed.

Cell cycle inhibitor tumor suppressor p21 was poorly expressed in the vaginal muscularis and epithelial layers of both *Fbln-5^-/-^* and WT mice. No significant differences were seen in the expression of p21 at any time point, though at 20 weeks there was a trend in which immunostaining in the *Fbln-5^-/-^* treatment group was higher compared to all groups (Table 1).

Expression of phosphorylated gamma histone 2A (γ-H2Ax) in vaginal tissues from WT and *Fbln-5^-/-^* mice did not differ significantly in either the control or D+Q groups (Table 2). SA-β-Gal activity remained relatively stable at 10, 12 and 20 weeks and also did not vary significantly between groups (Table 2). Cytokine concentration measurements from the multiplex analysis for ten markers, CCL-2, CCL-3, CCL-11, CCL-20, CXCL1, IL-Iβ, IL-6, IFN-γ, TNF-α and GM-CSF, did not change significantly over 10, 12 and 20 weeks and did not vary significantly between treatment and control arms in either the WT or *Fbln-5^-/-^* group, or between the WT and *Fbln-5^-/-^* group (Table 3).

**Table 2:** Percentage of mouse vaginal tissue stained for γ-H2Ax and SA-β-Gal immunofluorescence.

**Week**	**WT control**	**WT treatment**	***Fbln-5^-/-^* control**	***Fbl-n5^-/-^* treatment**	**p-value within WT**	**p-value within *Fbln-5^-/-^***	**p-value within WT and *Fbln-5^-/-^* D+Q groups**
γ-H2Ax							
10	10.54	6.50	3.51	2.46	>0.99[-25.16, 17.10]	>0.99 [-20.08, 22.19]	>0.99 [-24.13, 18.14)
12	4.97	6.88	2.10	14.38	>0.99 [-19.23, 23.04]	0.69 [-33.42, 8.85]	>0.99 [-25.91, 16.36]
20	17.21	3.61	5.96	3.07	0.55 [-34.73, 7.54]	>0.99 [-17.25, 23.12]	>0.99[-17.89, 22.58]
SA-β-Gal							
10	3.84	4.44	4.39	3.61	>0.99 [-3.74, 4.93]	>0.99 [-5.11, 3.55]	>0.99 [-5.16, 3.51]
12	4.91	3.34	2.87	3.61	0.97 [-5.74, 2.61]	>0.99 [-3.59, 5.08]	>0.99 [-4.06, 4.61]
20	3.56	3.57	3.72	4.38	>0.99 [-4.33, 4.34]	>0.99 [-3.30, 4.61]	0.99 [-3.14, 4.77]

Data are marginal means and p-values with 95% CI. WT control (n=27), WT treatment (n=27), *Fbln-5^-/-^* control (n=33), *Fbln-5^-/-^* treatment (n=30).

**Table 3:** Senescence-associated Secretory Phenotype (SASP) Cytokine markers evaluated by Bio-Rad MAGPIX Multiplex assay.

**SASP Cytokine**	***Fbln-5^-/-^* control**	***Fbln-5^-/-^* treatment**	**p-value**
CCL-2	49.30	54.90	>0.99 [-110.62, 121.78]
CCL-3	9.00	15.10	0.99 [-14.75, 27.01]
CCL-11	1209	2144	0.22 [-203.72, 2074.57]
CCL-20	8.80	13.64	0.85 [-4.57, 14.25]
CXCL1	97.00	121.20	>0.99 [-298.44, 346.82]
IL-Iβ	315.60	282.60	>0.99 [-959.61, 893.75]
IL-6	1.06	1.56	0.99 [-1.47, 2.46]
IFN-γ	4.50	6.23	0.998 [-5.90, 9.35]
TNF-α	5.96	7.03	> 0.99 [-6.20, 8.33]
GM-CSF	4.72	11.58	0.97 [-15.75, 29.47]

Data are marginal means and p-values with 95% CI. Representation of mean cytokine concentration (pg of cytokine/mg of protein) among *Fbln-5^-/-^* control and *Fbln-5^-/-^* treatment at 20 weeks of life.

### Evaluation of production of elastin and collagen

**Figure 4:**
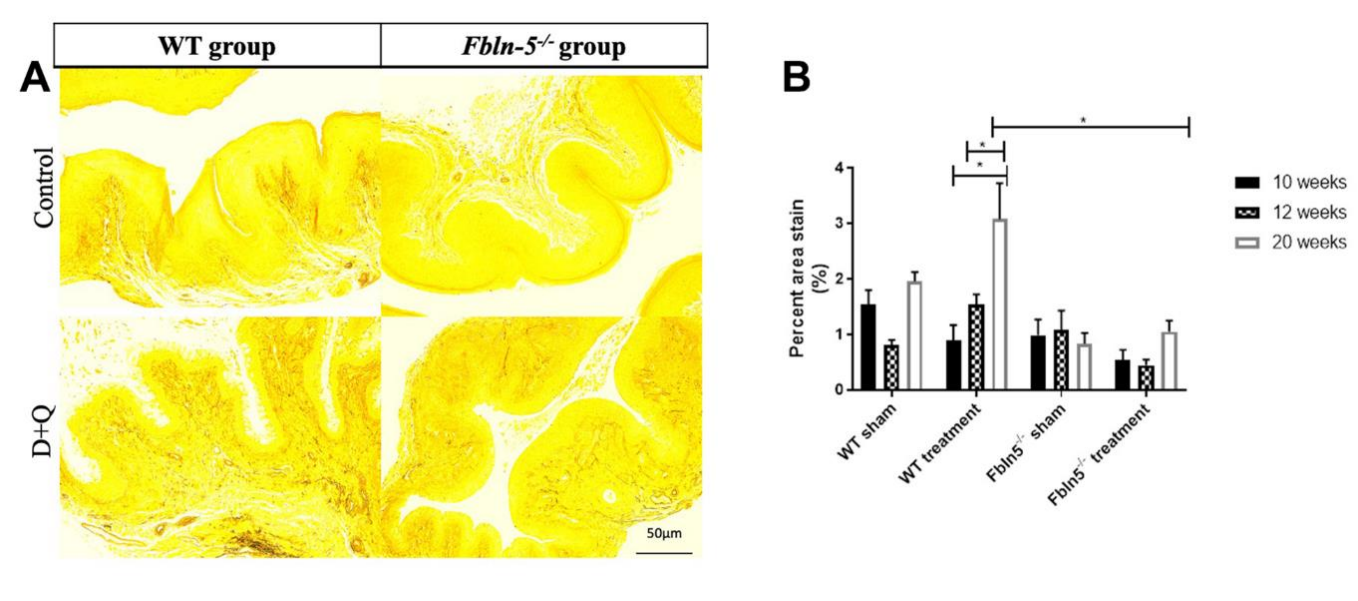
**Impact of serial dasatinib and quercetin (D+Q) injections on elastin content in the vaginal tissue of wild type (WT) and Fibulin-5 knockout (*Fbln-5^-/-^*) mice at 20 weeks of life**. Representative tissue sections from 20-week-old WT and *Fbln-5^-/-^* mice (**A**). Top panels correspond to WT and *Fbln-5^-/-^* mice in the control group and the bottom panels correspond to WT and *Fbln-5^-/-^* mice in the D+Q group. Quantification of vaginal tissue elastin content at 10, 12 and 20 weeks of life in WT control, WT treatment, *Fbln-5^-/-^* control and *Fbln-5^-/-^* treatment groups (**B**). Results are expressed as raw mean percent of total tissue ± SEM. 20x magnification.

**Figure 5:**
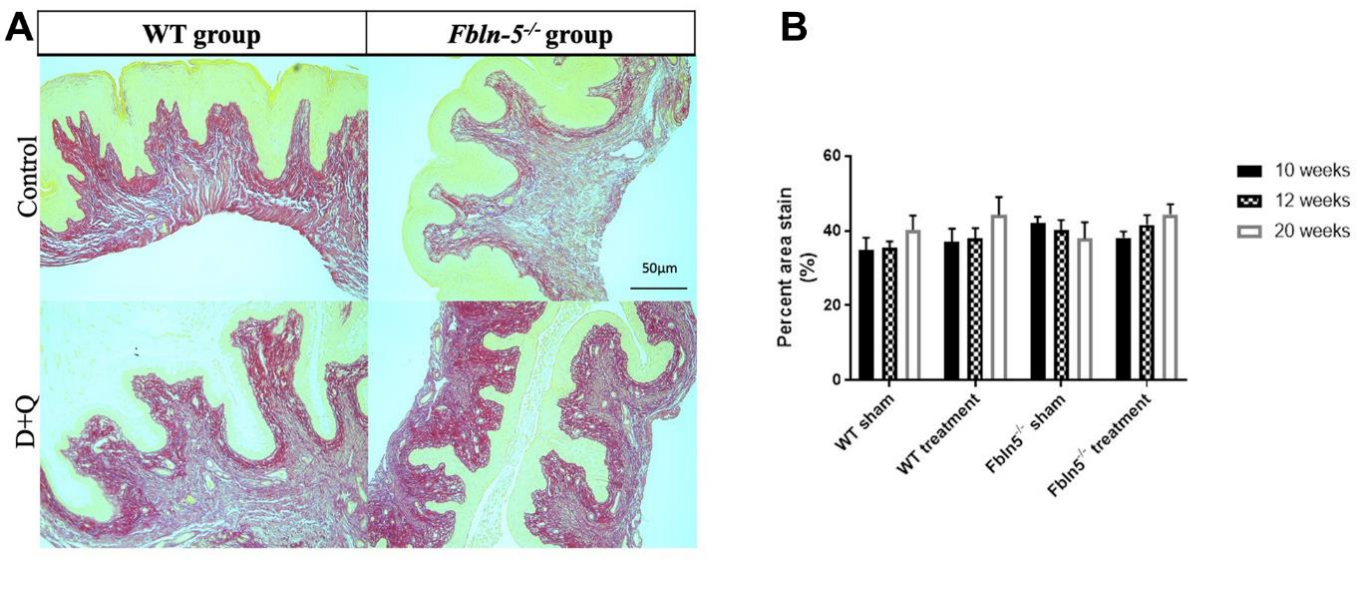
**Impact of serial dasatinib and quercetin (D+Q) injections on collagen content in the vaginal tissue of wild type (WT) and Fibulin-5 knockout (*Fbln-5^-/-^*) mice at 20 weeks of life.** Representative tissue sections from 20-week-old WT and *Fbln-5^-/-^* mice (**A**). Top panels correspond to WT and *Fbln-5^-/-^* mice in the control group and the bottom panels correspond to WT and *Fbln-5^-/-^* mice in the D+Q group. Quantification of vaginal tissue collagen content at 10, 12 and 20 weeks of life in WT control, WT treatment, *Fbln-5^-/-^* control and *Fbln-5^-/-^* treatment groups (**B**). Results are expressed as raw mean percent of total tissue ± SEM. 20x magnification.

To determine if serial injections of D+Q have an impact on the production of extracellular matrix proteins key in providing biomechanical support to vaginal tissue, histologic staining was used to measure elastin and collagen content within the vaginal tissue of WT and *Fbln-5^-/-^* mice. Elastin content increased from 12 to 20 weeks of age in the WT control, WT treatment and *Fbln-5^-/-^* treatment groups, and declined in only the *Fbln-5^-/-^* control group. At 20 weeks of age, elastin content was 0.84% of vaginal tissue in *Fbln-5^-/-^* control mice compared to 1.04% in *Fbln-5^-/-^* treatment mice, though this difference did not reach statistical significance (Figure 4). Collagen content was evaluated by picrosirius red staining. Collagen content remained relatively stable over time in both WT and *Fbln-5^-/-^* mice and did not significantly differ between treatment and control groups (Figure 5).

## DISCUSSION

### Impact of serial D+Q injections on clinical development of pelvic organ prolapse

In our clinical assessment of the impact of serial D+Q injections on development of pelvic organ prolapse, no significant differences were seen in vaginal bulge height in either WT or *Fbln-5^-/-^* mice receiving D+Q injections compared to control animals. Significant changes did arise in the *Fbln-5^-/-^* mice treatment group, in which PBL became significantly longer than that of *Fbln-5^-/-^* control and WT control group mice beginning at 18 weeks of age. Importantly, just one animal in the *Fbln-5^-/-^* treatment arm had Stage 2 prolapse, with the remainder of mice having predominantly Stage 0 prolapse. Prior studies have shown that >90% of *Fbln-5^-/-^* mice will develop prolapse by 24 weeks of age [6].

In this study, MOP-Q measurements were performed up to 20 weeks of age when development of prolapse may not have yet progressed to advanced stages, defined as Stage 2 or greater. Long-term follow up to 24 weeks of age and beyond is needed to make further conclusions on the impact of D+Q of injections on POP development.

### Impact of serial D+Q injections on senescence markers

Expression of both p53 and p16 were downregulated in *Fbln-5^-/-^* mice receiving D+Q injections compared to control animals, with the latter reaching statistical significance at 20 weeks of age. The p53/p21 pathway and p16/retinoblastoma pathway both represent key modulators of cell cycle inhibition and tumor suppression. Activation of these pathways is integral in normal cell turnover, and in young animals, activation of cell cycle inhibition promotes cellular senescence that is preventative of proliferation of damaged cells [10]. In aging animals, accumulation of senescent cells promotes deleterious cellular changes that lead to activation of SASP components including pro-inflammatory cytokines and extracellular matrix proteases capable of inducing tissue damage and fibrosis [18, 19]. This transition from protective to deleterious outcomes is dependent on the severity and duration of stress stimuli promoting senescence, such as telomere shortening and exposure to reactive oxygen species that occurs with age [20]. Prior studies have shown that p53-mediated pathways are important for the initiation of cellular senescence and p16-mediated pathways are important for promoting a sustained state of senescence [21].

In this study, absence of concomitant changes in expression of SASP cytokines and SA-β-Gal makes it difficult to determine if increased expression of p53 and p16 in *Fbln-5^-/-^* control mice were in fact due to transient activation of cell cycle inhibitors, or as a result of sustained cellular senescence, as activation of cell cycle inhibitors alone is not specific to senescence. Despite this, it is biologically plausible that differences in p53 and p16 expression may be a result of the senolytic activity of D+Q injections. Dasatinib, a tyrosine kinase inhibitor, and quercetin, a naturally occurring flavonoid that binds to BCL-2, both inhibit the PI3K/AKT pathway which alters pro-survival pathways in senescent cells, including downstream effects on cell cycle inhibition pathways [22]. Long-term follow up in older animals evaluating clinical development of prolapse and sustained cellular changes associated with cellular senescence will help to confirm the impact of D+Q on alterations in cell cycle inhibitor expression.

Expression of γ-H2Ax and SA-β-Gal remained relatively stable and did not change significantly in both WT and *Fbln-5^-/-^* control and treatment groups at all time points analyzed. γ-H2Ax serves as a marker of DNA damage and its expression is known to be altered after exposure to vaginal tissue trauma in both mouse and rat models [8, 9]. It is possible the absence of a traumatic event, such as vaginal birth, led to stable levels of γ-H2Ax expression in all groups of WT and *Fbln-5^-/-^* mice. Overexpression and accumulation of endogenous lysosomal beta-galactosidase occurs specifically in senescent cells [23]. In young healthy WT mice and *Fbln-5^-/-^* mice without significant prolapse, senescent cells may make up a small portion of vaginal tissue cells, resulting in overall low SA-β-Gal activity.

Similarly, multiplex analysis of SASP components did not demonstrate significant variation in concentration of ten SASP cytokines among both WT and *Fbln-5^-/-^* control and treatment groups. SASP cytokines are activated in sustained states of cellular senescence and work in both an autocrine and paracrine fashion to maintain and promote senescence within affected and surrounding cells [24]. Additionally, different disease states are associated with unique SASP components [25]. Vaginal distention in *Fbln-5^-/-^* mice has been shown to induce activity of proteases (MMP2 and MMP9) in vaginal tissue, but increased production of other SASP cytokines unique to pelvic organ prolapse in mice has not yet been completely elucidated [7, 8]. Future studies are needed to determine which SASP markers are consistently elevated in states of clinically advanced prolapse.

### Impact of serial D+Q injections on production of extracellular matrix proteins

Increased elastin production in *Fbln-5^-/-^* mice receiving D+Q injections was seen at 20 weeks of age compared to *Fbln-5^-/-^* mice in the control group, though this was not statistically significant. This represents an important finding as *Fbln-5^-/-^* mice develop prolapse due to genetic deficiencies in elastin fiber assembly, therefore therapeutic modalities targeting this deficit may play a role in the prevention and treatment of prolapse. Elastin fiber content in *Fbln-5^-/-^* mice receiving D+Q injections did not reach equal levels of elastin fiber content found in WT mice vaginal tissue, but the possibility for further improvement in elastin production should be explored in mice 24 weeks of age and older to determine if this trend is maintained.

### Strengths and limitations

This study has several strengths including the use of a combination of senolytic agents, D+Q, which has been shown to reduce senescent cell burden in multiple disease states such as chronic kidney disease and pulmonary fibrosis [16, 17]. Multiple methods were utilized to evaluate for a range of different senescence markers including immunostaining for common cell cycle inhibitors and γ-H2Ax, a multiplex analysis of ten well established SASP markers, and analysis of SA-β-Gal activity and extracellular matrix components. Finally, robust comparison groups were utilized which included both *Fbln-5^-/-^* controls and corresponding WT comparison groups. One major limitation of this study is follow-up limited to 20 weeks of age in both WT and *Fbln-5^-/-^* mice. The great majority of *Fbln-5^-/-^* mice will develop prolapse by 24 weeks of age, therefore, longer periods of follow up will allow for enhanced evaluation of clinically significant prolapse and associated cellular changes. Use of *Fbln-5^-/-^* mice genetically pre-disposed to prolapse due to elastin fiber deficiency is a potential limitation as the mechanism for prolapse development in these mice may differ from senescence associated with age alone or tissue trauma. Our study exclusively evaluated vaginal tissue and did not examine other anatomic structures associated with pelvic floor support that play a role in the development of prolapse.

### Conclusions and future directions

This study represents one of the first to evaluate the impact of senolytic agents D+Q on the clinical development of pelvic organ prolapse and expression of proteins associated with cellular senescence in a mouse model. To date, assessment of the impact of senolytic agents in vaginal tissue is relatively limited with just one study reporting *in vitro* outcomes of D+Q injections in rat vaginal fibroblasts exposed to etoposide [9]. Alteration in expression of cell cycle inhibitors plays an important role in the initiation and maintenance of cellular senescence. Our findings of downregulation of p53 and p16, as well as increased elastin content, in vaginal tissue of *Fbln-5^-/-^* mice receiving D+Q injections demonstrate the potential for senolytic agents to target early changes associated with cellular senescence, which may ultimately contribute to the development of pelvic organ prolapse. Further studies are needed to confirm if this trend is maintained as mice age and go on to develop more advanced prolapse. Both the dose and timing of D+Q administration will also require further evaluation as early administration may be detrimental to normal protective cell cycle inhibition seen in young mice, whereas late administration may miss a critical timeframe in which cellular changes associated with the development of prolapse may be inhibited. The route of administration of senolytic agents also deserves further validation to confirm which methods result in the highest levels of drug bioavailability within our target tissue, while minimizing adverse side effects in tissues outside the pelvic floor. Finally, this study targeted cellular changes associated with prolapse related to age. Future studies should evaluate the impact of senolytic agents on other risk factors associated with the development of pelvic organ prolapse such as pelvic floor trauma associated with vaginal delivery. Findings of such studies may potentially have a significant clinical impact on recommendations for the prevention of pelvic organ prolapse in the large patient population of reproductive-aged women.

## MATERIALS AND METHODS

Female mice (wild-type (WT), n=54; *Fbln-5^-/-^* n=63) were studied and euthanized in accordance with standards of humane animal care described by the National Institutes of Health Guide for the Care and Use of Laboratory Animals, using protocols approved by the Institutional Animal Care and Use Committee of the University of Texas Southwestern Medical Center. Animals were housed under 12:12-hours light-dark cycle (lights on 6:00 AM, lights off 6:00 PM) at 22° C.

At four weeks of age, mice were randomized to either a control group (vehicle injection) or treatment group (D+Q). Oral gavage injections of the treatment regimen dasatinib (Sigma-Aldrich, SML2589, 5mg/kg, USA) and quercetin (Sigma-Aldrich, Q4951, 50mg/kg) or the control regimen 10% PEG400 (BroadPharm, 25322-68-3, 200 μL, USA) were administered at 4, 5, 6, 7 and 8 weeks of life. Mouse pelvic organ prolapse quantification system (MOP-Q) measurements were obtained weekly by two authors (ET and HS) [6]. Vaginal tissue was harvested at 10, 12 and 20 weeks. *Fbln-5^-/-^* mice develop POP as early as several weeks after sexual maturity (10-12wk) and severity of prolapse increases rapidly with age with >90% developing prolapse by 6 months [6]. Tissue harvesting timepoints were designed to detect changes occurring prior to and early in the development of prolapse.

Animals were euthanized and after disarticulation of the pubic symphysis, uterine horns together with the bladder, cervix, and vagina were dissected to the perineal skin. The vaginal dissection extended to the connective tissue, suspending the vaginal wall to the pubocaudalis. Using microdissection, the cervix, uterus, bladder, urethra and perineal skin were removed from the vagina. The vaginal tissue was cut into four equally sized rings. The distal ring was used for cytokine analysis. The proximal ring was placed in 10% formalin (Sigma-Aldrich, St. Louis, MO, USA) for 24 hours and then transferred to 70% ethanol for further sectioning and staining. The mid-vaginal rings were placed in optimal cutting temperature compound (OCT) (Fisher Healthcare, Houston, TX, USA) for sectioning and immunofluorescence staining, and into phosphate buffered saline 1X (PBS) (Sigma-Aldrich) at room temperature for senescence-associated-β-galactosidase (SA-β-Gal) activity analysis.

### Immunofluorescence

Vaginal tissue rings placed in OCT and frozen on dry ice beds immediately after dissection were stored at – 20°C. These tissue blocks were sectioned with the cryostat (Leica CM1950, Nussloch, Germany) at 15 μm thickness with up to four sections per slide. Four groups of immunofluorescence stains were used per animal. The slides were fixed with 4% PFA in PBS 1X for 10 minutes and incubated with 10% normal goat serum (Fisher Scientific, Hampton, NH, USA) and 0.05% Triton 100x (Mallinckrodt, Paris, ON, Canada) for 2 hours. The slides were then stained with primary monoclonal antibodies of mouse (ms) Anti-p16 (Abcam, 54210, USA), rabbit (rb) Anti-p21 (Abcam, 109199), ms Anti-p53 (Abcam, 26) and ms Anti-γ-H2Ax (Abcam, 22551) in 1% BSA at 4°C for 20 hours. Slides were then incubated with secondary antibodies of Goat Anti-rb IgG AlexaFlour (AF) 488 (Life Technologies, A11034, USA) and Goat Anti-ms AF594 (Life Technologies, A11001) in 1% BSA for 1 hour at room temperature. DAPI (Fisher Scientific, USA) was applied to slides that were then mounted with cover slips. Negative slides without primary antibodies and double negative slides without primary and secondary antibodies served as controls.

Confocal laser scanning microscopy (Zeiss LSM880 Airyscan) was used to examine and image immunostained slides at 20x magnification. Three randomly selected regions were imaged per tissue sample. These vaginal cross-sectional images were evaluated with Fiji Image-J software by authors ET and HS. Basal threshold of fluorescence was used for quantification of signal area. This was done by selecting a pre-existing threshold method that was most representative of the stained area. Thresholded images first captured the total tissue size and then the target protein expression (p16, p21, p53, γ-H2Ax). Results were analyzed as area fraction (percentage of pixels) of each above antibody within the total tissue area.

### Cytokine analysis

The distal vaginal ring tissue from each mouse was flash frozen using liquid nitrogen and stored at -80°C. Samples were thawed and processed for protein lysate using the Bio-Plex Cell Lysis Kit (Bio-Rad, Hercules, CA, USA) according to the kit protocol. The sample protein concentrations were measured by the bicinchoninic acid (BCA) Protein Assay Kit (Thermo Fischer #23225 and 23227).

The inflammatory cytokines CCL-2, CCL-3, IL-Iβ, IFN-γ, TNF-α, CCL-11, GM-CSF, IL-6, CCL-20, and CXCL1 were measured in pg/mL using the Bio-Plex Pro Mouse Chemokine 10plx on the MAGPIX Multiplex Reader (Luminex, Austin, TX, USA). Cytokine concentration results were normalized using the total sample protein concentration. The samples were analyzed in duplicate. Samples with an average bead count below 50 or with cytokine concentrations that were widely variable (more than 10% from the mean) were excluded. Final cytokine concentrations are reported as pg of cytokine per mg of protein.

### Senescence-associated-β-galactosidase

Vaginal tissue was washed in PBS 1X, then histochemical staining to identify SA-β-Gal was performed using a commercially available X-gal staining kit (Abcam, Cambridge, MA, USA) adapted from a previously published protocol [19, 26]. Tissue was incubated in fixative solution with agitation for 30 minutes, then washed with detergent solution for 20 min under agitation for a total of 3 washes. The tissue was washed once with PBS then incubated in X-gal staining solution for 24 hours at 37°C. The reagents for X-gal were prepared per the manufacturer’s instructions including staining solution, staining supplement and X-gal. Mouse kidney tissue was used as a positive control. After incubation, tissue samples were removed from X-gal, washed with PBS, embedded in OCT and stored at -20°C. Tissue blocks were sectioned at 15 μm thickness using a cryostat with 6-8 sections per slide. Aqua-Mount (Fisher Scientific) was applied to slides that were then mounted with cover slips. Tissue slides were examined and imaged using light microscopy at 20x magnification (Leica, Model DMi8, Nussloch, Germany). Fiji Image-J software was used to examine the amount of blue-colored stain present in each tissue. This was done by manually setting the threshold to visible tissue staining via brightfield microscopy for each image first to capture the total tissue area and then to capture the amount of blue stain within the tissue. The pixel counts of each were measured and used to calculate the percentage of blue-colored X-gal product stain within the tissue. This was repeated for up to 12 tissue samples per treatment or sham group.

### Histologic analysis

Vaginal tissue was fixed in 10% formalin solution for 24 hours and then transferred to 70% ethanol. Tissues were then embedded in paraffin and sectioned further using standard methods, then stained with elastin, picrosirius red, and hematoxylin and eosin (H&E). Tissue slides were then examined using light microscopy and Leica camera images were captured at 10x magnification. Three randomly selected tissue regions were captured per tissue sample for all slides. Fiji Image-J software was then used to examine the amount of elastin and picrosirius red present in each tissue. This was done by selecting a pre-existing threshold method that was most representative of the stained area. Thresholded images captured the total tissue area and then the amount of elastin fibers or picrosirius red within the tissue. The pixel counts of each were measured and used to calculate a percentage of the stained area versus the total tissue area.

### Statistical analysis

Power analysis was performed using our previously published data on *Fbln-5^-/-^* and markers of cellular senescence [8]. Four animals in each group were required for 95% power to detect a difference at p-value of < 0.05. Additional animals were included to allow for potential loss or problems with tissue handling/dissection. Each outcome measure was assessed using a generalized linear mixed effects model. The correlation structure was estimated with no restrictions. The fixed effects in each model were mouse genotype and treatment or control arm, with time as a random effect. Two-way interactions were estimated, and the marginal means were reported. Pairwise comparisons were made, and p-values were adjusted for multiplicity using the Tukey method. The p-values were reported alongside 95% confidence intervals. Statistical significance is indicated by p < 0.05. Statistical analysis was performed in R (Vienna, Austria) [27].
